# Hydrangea‐Like CuS with Irreversible Amorphization Transition for High‐Performance Sodium‐Ion Storage

**DOI:** 10.1002/advs.201903279

**Published:** 2020-04-08

**Authors:** Zu‐Guang Yang, Zhen‐Guo Wu, Wei‐Bo Hua, Yao Xiao, Gong‐Ke Wang, Yu‐Xia Liu, Chun‐Jin Wu, Yong‐Chun Li, Ben‐He Zhong, Wei Xiang, Yan‐Jun Zhong, Xiao‐Dong Guo

**Affiliations:** ^1^ School of Chemical Engineering Sichuan University Chengdu 610065 P. R. China; ^2^ Institute for Applied Materials (IAM) Karlsruhe Institute of Technology (KIT) Hermann‐von‐Helmholtz‐Platz 1 Eggenstein‐Leopoldshafen 76344 Germany; ^3^ School of Materials Science and Engineering Henan Normal University Xinxiang 453007 P. R. China; ^4^ The Key Laboratory of Life‐Organic Analysis Key Laboratory of Pharmaceutical Intermediates and Analysis of Natural Medicine School of Chemistry and Chemical Engineering Qufu Normal University Qufu 273165 P. R. China; ^5^ College of Materials and Chemistry &Chemical Engineering Chengdu University of Technology Chengdu 610059 P. R. China

**Keywords:** hydrangea‐like CuS, in situ synchrotron radiation diffraction, irreversible amorphization, sodium‐ion batteries

## Abstract

Metal sulfides have been intensively investigated for efficient sodium‐ion storage due to their high capacity. However, the mechanisms behind the reaction pathways and phase transformation are still unclear. Moreover, the effects of designed nanostructure on the electrochemical behaviors are rarely reported. Herein, a hydrangea‐like CuS microsphere is prepared via a facile synthetic method and displays significantly enhanced rate and cycle performance. Unlike the traditional intercalation and conversion reactions, an irreversible amorphization process is evidenced and elucidated with the help of in situ high‐resolution synchrotron radiation diffraction analyses, and transmission electron microscopy. The oriented (006) crystal plane growth of the primary CuS nanosheets provide more channels and adsorption sites for Na ions intercalation and the resultant low overpotential is beneficial for the amorphous Cu‐S cluster, which is consistent with the density functional theory calculation. This study can offer new insights into the correlation between the atomic‐scale phase transformation and macro‐scale nanostructure design and open a new principle for the electrode materials' design.

## Graphical abstract

A hydrangea‐like CuS microsphere with high geometrical symmetry and oriented (006) crystal plane growth is successfully constructed through a facile synthetic route. The oriented (006) crystal plane growth of the primary CuS nanosheets provide more channels and adsorption sites for Na ions intercalation, and the resultant low overpotential is beneficial for the amorphous Cu‐S cluster.

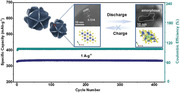

Large‐scale electrochemical energy storage systems in a cheap and efficient way have attracted widespread attention,^[^ ^1^, ^2^ ^]^ and sodium ion batteries are one of the promising devices for large‐scale energy storage.^[^ ^3^, ^4^, ^5^ ^]^ In the past decades, much effort has been devoted to developing different materials and strategies for Na‐ion battery and other energy storage materials.^[^ ^6^, ^7^, ^8^, ^9^ ^]^ Owing to the distinction on standard electrode potential (−2.71 V vs −3.02 V for Na^+^ vs Li^+^) and ion radius (1.02 vs 0.76 Å for Na^+^ vs Li^+^), obvious differences about diffusion kinetics and migration barrier energy have a profound influence on the reaction pathways and phase transformation during charge–discharge process.^[^ ^10^, ^11^, ^12^ ^]^ The Na^+^ diffusion pathways and reversible structural transformation in the intercalation reaction progresses have been systematically studied in cathode materials,^[^ ^13^, ^14^, ^15^, ^16^, ^17^, ^18^, ^19^, ^20^ ^]^ and the emergence of an insertion process, a conversion reaction, or an alloying–dealloying reaction during the electrochemical process is determined with chalcogenide, which is also crucial to reaction pathways and phase transformation.^[^ ^21^, ^22^, ^23^, ^24^, ^25^, ^26^, ^27^, ^28^, ^29^, ^30^ ^]^

Recently, the traditional intercalation and conversion reactions have been extensively investigated in Cu*_x_*S system. CuS would react with Na^+^ to form intermediate Na_α_Cu_β_S_γ_ with three distinct phases in a following sequence, Na(CuS)_4_, Na_7_(Cu_6_S_5_)_2_, and Na_3_(CuS)_4_, and then accompany with Na_2_S phase and metallic copper formation,^[^ ^31^, ^32^ ^]^ and the CuS structure can be rebuild with depleting Na_2_S and metallic copper consequently.^[^ ^33^, ^34^, ^35^ ^]^ In addition, Park et al. found that Cu_1.8_S shows an intercalation/deintercalation reaction mechanism without irreversible phase transformation, but most of metal sulfides experience a conversion reaction mechanism,^[^ ^36^ ^]^ and there are some similar researches to CuS materials for sodium ion storage.^[^ ^37^, ^38^ ^]^ According to the latest report, nitrogen‐doped carbon‐coated Cu_9_S_5_ bullet‐like hollow particles and hierarchical CuS@CoS_2_ double‐shelled nanoboxes have been prepared by morphology design and nanostructure engineering and both have superior rate capability and long cycle life.^[^ ^39^, ^40^ ^]^ However, behind the reaction mechanisms there still exist some unclear issues related to the reaction pathways and phase transformation. Moreover, the effects of designed nanostructure on the electrochemical behaviors are rarely reported. Consequently, the relation between designed morphology and electrochemical behaviors and the sodium‐ion storage mechanism behind require a comprehensive in‐depth study, which would share some clues to understanding the relation between the designed morphology and electrochemical behaviors of other sulfides.

Herein, we fabricate hydrangea‐like CuS microsphere with high geometrical symmetry and oriented (006) crystal plane growth, which exhibits superior rate capability and excellent cycling stability. A reversible capacity of 335 mAh g^−1^ is maintained after more than 400 cycles without capacity fading. Unlike the traditional intercalation and conversion reactions, an irreversible amorphization process was evidenced and elucidated through in situ synchrotron radiation diffraction (SRD) and transmission electron microscopy (TEM) analyses. The oriented (006) crystal plane growth of the primary CuS nanosheets provide more channels and adsorption sites for Na ions intercalation, and the resultant low overpotential is beneficial for the amorphous Cu‐S cluster, which is consistent with the density functional theory (DFT) calculation. This study may provide a new clue for understanding the correlation between the atomic‐scale phase transformation and macro‐scale nanostructure design.

Hydrangea‐like CuS microspheres were prepared by a facile hydrothermal method. Copper(II) nitrate hydrate and sulfur powder were dispersed in the anhydrous ethanol and polyvinyl pyrrolidone (PVP) was used as surfactant, and the material without PVP had uneven sphere morphology (Figure S1, Supporting Information). To investigate the growth mechanism of hydrangea‐like CuS microsphere, the morphologies of intermediate products at different reaction temperatures and various reaction times were observed by scanning electron microscope (SEM). The morphologies of materials were obviously different under different reaction temperatures (Figure S2, Supporting Information). It could be confirmed that the morphology was uniform at 180 °C. As shown in **Figure** **1**b–f, the reaction time has a great influence on the growth status (such as shape and size) of the products. We have found the nanospheres with irregular shapes at earlier reaction stage. As the reaction time increased, the nanospheres had homogenous size and the rough surface faded away due to the rough surface with numerous high surface energy sites for oriented nanocrystal growth, and the irregular nanoplates evoluted and transformed into the microdisks (Figure 1b–d) because the irregular nanospheres gradually dissolved at low surface energy sites and recrystallized along high‐energy sites.^[^ ^41^ ^]^ At the reaction time of 8 h, regular CuS microspheres with a diameter of ≈1.5 µm consisting of some converged microplates have formed. The mass diffusion and Ostwald ripening process have played an important role in the formation of hydrangea‐like CuS microsphere.^[^ ^42^ ^]^ In Figure 1e, the thickness of the microplates is increased at the reaction time of 12 h. This growth process may be associated with the preferential growth direction and stacking fault planes.^[^ ^43^ ^]^ With further extension of the reaction time at 18 h, the microplates have cracked because of the stress induced, as shown in Figure 1f. From Figure S3, Supporting Information, we can confirm that the samples prepared with different conditions belong to the same hexagonal CuS structure, matching well with the standard values (PDF#06‐0464).

**Figure 1:**
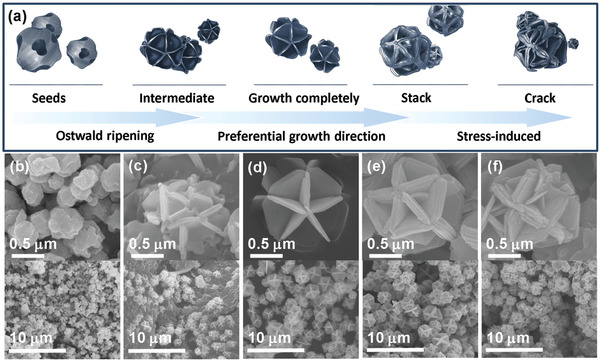
a) Schematic illustration of the formation mechanism of CuS spheres. SEM images of CuS prepared at 180 °C with different reaction times: b) 1 h, c) 4 h, d) 8 h, e) 12 h, and f) 18 h.

**Figure** **2**a,b shows SEM images of the hydrangea‐like CuS with high geometrical symmetry. The hydrangea‐like CuS have a homogeneous size distribution with an average diameter of about 1.5 µm, corresponding to hydrangea model as depicted in Figure 2c. The TEM, HRTEM, and SAED of nanoplates are presented in Figure 2d. Two groups of lattice fringes (102) and (01‐2) have the same interplanar distance of 3.04 Å and form an angle of 73°. The corresponding SAED pattern presents an array of dots with single‐crystalline, which can be indexed to the (‐11‐4), (01‐2), and (102) crystal planes of CuS. Figure 2e displays a TEM image of the side in CuS nanoplates. The HRTEM and corresponding FFT pattern of the side in CuS nanoplates are also displayed in Figure 2e. A lattice spacing of 2.72 Å could be observed corresponding to the lattice spacing of (006) plane in HRTEM. The corresponding FFT pattern can be indexed to the (201), (207), and (006) crystal planes of CuS. Figure 2f shows TEM images of the hydrangea‐like CuS and the corresponding EDS elemental mapping images for Cu and S elements, indicating that Cu (blue) and S (red) elements have homogeneous distribution throughout the overall region. The X‐ray diffractometer (XRD) pattern of the hydrangea‐like CuS analyzed by Rietveld refinement is presented in Figure 2g. All diffraction peaks of the sample perfectly exhibit a hexagonal CuS structure (space group: *P*63/*mmc*) with lattice parameters *a* = *b* = 3.792 Å and *c* = 16.344 Å, matching well with the standard values (PDF#06‐0464). The peaks at 27.1°, 27.7°, 29.2°, 31.7°, 32.8°, 38.8°, 43.1°, 47.9°, 52.7°, and 59.3° corresponded to the crystal planes of (100), (101), (102), (103), (006), (105), (106), (110), (108), and (116), respectively. Figure 2h displays the crystal structures of typical layered CuS viewed along *b* axis and *c* axis. All research results in the crystal structure confirmed that pure hexagonal phase CuS with high crystallinity is prepared. The XPS was carried out to further investigate the surface composition and element chemical state of hydrangea‐like CuS. Figure 2i shows the Cu 2p high‐resolution XPS spectrum for hydrangea‐like CuS. Two peaks at 932.5 and 952.4 eV could be assigned to Cu^2+^ 2p_3/2_ and Cu^2+^ 2p_1/2_, respectively.^[^ ^44^ ^]^ As for the S 2p high‐resolution spectrum in Figure 2j, the binding energies centered at 161.83 and 162.9 eV are derived from S 2p_3/2_ and S 2p_1/2_ of S^2−^.^[^ ^45^ ^]^ It is indicated that the Cu^2+^ and S^2−^ present in the hydrangea‐like CuS.

**Figure 2:**
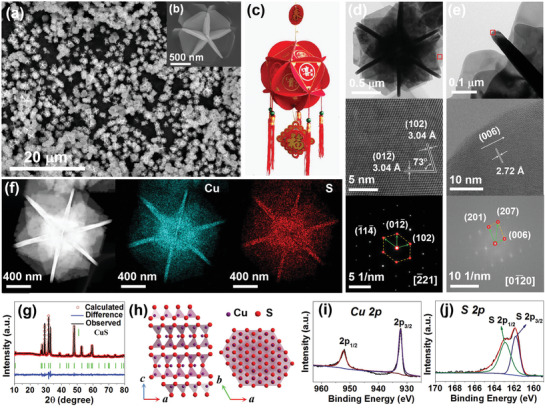
a,b) SEM image and c) corresponding model. d) TEM image at a low magnification, high‐resolution TEM image, and selected‐area electron diffraction pattern (SAED). e) TEM image at a low magnification, high‐resolution TEM image, and the fast Fourier transform (FFT) pattern. f) HAADF‐STEM image and the corresponding elemental mapping images for Cu element (blue) and S element (red). g) Powder XRD patterns and Rietveld refinement plots. h) Crystal structure viewed along *b* axis (left) and *c* axis (right). High‐resolution X‐ray photoelectron spectra (XPS) of the CuS: i) Cu 2p, j) S 2p.

Coin‐type cell was applied to evaluate the sodium‐ion storage properties of hydrangea‐like CuS. The rate performance of CuS electrode is shown in **Figure** **3**a. It can be observed that the CuS electrode displays average capacities of 350, 344, 341, 336, 314, and 238 mAh⋅g^−1^ at current densities of 0.1, 0.2, 0.5, 1.0, 2.0, and 5.0 A⋅g^−1^, respectively. A capacity of 349 mAh⋅g^−1^ was maintained after the current density returned to 0.1 A g^−1^, indicating the prominent reversibility of the hydrangea‐like CuS electrode. The corresponding galvanostatic discharge–charge profiles at the different current densities are displayed in Figure 3b with 80% initial coulombic efficiency. This initial irreversible capacity was mainly caused by the irreversible formation of solid–electrolyte interface film and the electrolyte decomposition.^[^ ^46^, ^47^ ^]^ To better understand their sodium‐ion storage mechanism and kinetics, sweep‐rate‐dependent cyclic voltammetry (CV) experiments were performed. The CV curves of CuS electrode at different scan rates from 0.1 to 2 mV s^−1^ are presented in Figure 3c after cell activation, and the galvanostatic discharge/charge (GDC) profiles in the first three cycles together with the differential capacity (dQ/dV) curves in Figure S4, Supporting Information. It could be confirmed that the twostep reaction has occurred in the active materials. In Figure 3c, there are obvious reduction/oxidation peaks in CV curves. Two reduction peaks at around 1.5 and 0.8 V relate to two‐step phase transition triggered by the intercalation of Na^+^ into CuS structure, and the two oxidation peaks at around 1.7 and 2.1 V contributed to the deintercalation of Na^+^ from Na*_x_*Cu*_y_*S*_z_*.^[^ ^48^, ^49^ ^]^ The relationship between measured current (*i*) and scan rate (*v*) from the CV curves conform to the equation *i* = *av^b^*, where *a* and *b* both are constants. It could be used to qualitatively analyze the degree of capacitive effect. The *b* value is between 0.5 and 1.0 and it could be confirmed by the slope of the log *i* versus log *v* plot. Generally, *b* is close to 0.5 representing a diffusion‐controlled process, and *b* approaches 1.0 meaning a surface capacitance‐dominated process.^[^ ^50^, ^51^ ^]^ Four peak currents (*i*) and scan rates (*v*) were selected to analyze the sodium‐ion storage kinetics processes in Figure 3d. Figure 3d displays the linear relationship between log(*i*) and log(ν) according to the following equation: log(*i*) = blog(*v*) + log(*a*). The *b* values of four peaks are 0.82, 0.75, 0.91, 0.93, respectively, which implies that the pseudocapacitive contribution dominates the capacity contribution of hydrangea‐like CuS electrode. To be more accurate, based on the equation of *i* (V) = *k*_1_ *v* + *k*_2_ *v* ^1/2^ (where *k*_1_ *v* and *k*_2_ *v* ^1/2^ represent the capacitive current and the diffusion controlled current, respectively), the total capacitive contribution can be separated into capacitive‐controlled contribution *k*_1_ *v* and diffusion‐controlled contribution *k*_2_ *v*_1/2_.^[^ ^52^ ^]^ By determining the *k*_1_ and *k*_2_ constants, a dominating pseudocapacitive contribution of 84.4% at 0.5 mV s^−1^ was gained as shown in Figure 3e. The contribution of capacitance increases from 78.9% to 94.9% with the sweeping rate ranging from 0.1 to 2.0 mV s^−1^ as shown in Figure 3f. The results indicate that the pseudocapacitive contribution the advantages in overall charge–discharge processes. And the occurrence of pseudocapacitance is beneficial to enhance the rate performance. The discharge/charge (GDC) profile after 400 cycles is displayed in Figure S4c, Supporting Information, and The XRD and TEM data for the electrodes after 400 cycles have been supplied as in Figure S5, Supporting Information. According to XRD data, it could be confirmed that the Cu_2_S have been formed for the electrodes after 400 cycles. Although the crystal structure of the material has changed, it could be observed from the TEM image that the material could keep its original morphology after 400 cycles. This result indicates that the pseudocapacitive contribution for sodium‐ion storage could be maintained in the cycling process leading to the superior electrochemical performance. To further verify the cycle performance of the hydrangea‐like CuS, the cycling test under high current density of 1 A g^−1^ was conducted. Figure 3g shows the superior cyclic performance of hydrangea‐like CuS at a current density of 1 A g^−1^ and a reversible capacity of 335 mAh⋅g^−1^ is maintained after more than 400 cycles without capacity fading. A short comparison of the electrochemical performance with the previous reports is presented in Table S1, Supporting Information.

**Figure 3:**
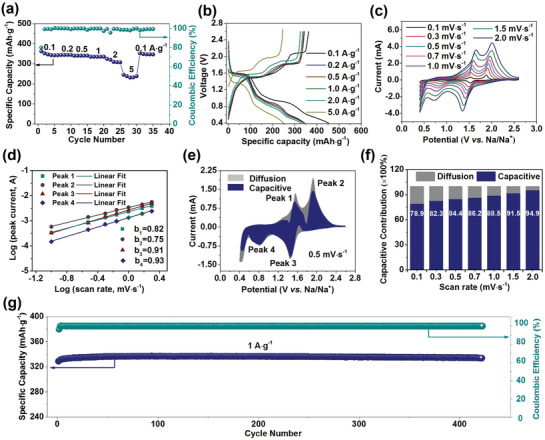
a) Rate capability of the CuS electrode at various current densities from 0.1 to 5 A g^−1^. b) First galvanostatic charge/discharge curves versus specific capacity of the CuS electrode at the different current densities. c) CV curves of the CuS electrode at different sweep rates from 0.1 mV s^−1^ to 2 mV s^−1^. d) Linear fitting of the log(*i*) versus log(*v*) plots at different oxidized and reduced state corresponding to CV curves in (c). e) Capacitive and diffusion‐controlled contribution to the overall charge storage of the CuS electrode at a scan rate of 0.5 mV s^−1^. f) Contribution ratio of capacitive and diffusion‐controlled behavior at different sweep rates of CuS electrode. g) Cycling performances and the corresponding coulombic efficiency of CuS electrode at a current density of 1 A g^−1^.

To clearly understand the sodium‐ion storage mechanisms of hydrangea‐like CuS during discharge/charge process between 0.4 and 2.6 V, in situ high‐resolution SRD measurement is performed in the first and second cycle galvanostatic profiles, as shown in **Figure** **4**a. The as‐fabricated CuS electrode shows the characteristic (102), (103), (006), (110), (108), and (116) peaks at 4°, 4.35°, 4.45°, 6.45°, 7.05°, and 7.85°, respectively, well matched with that given in Figure 2g. The peaks at 3° and 5.8° could be assigned to carbon paper and impurity imported by test condition. The change of characteristic peaks could be analyzed to study the structural transition of the material during sodium‐ion storage process, as presented in Figure 4b. To further investigate the sodium‐ion storage mechanism, TEM observation was also performed at different charge–discharge state (c–h blue point on charge–discharge curves in Figure 4a). In Figure 4c–h, HRTEM and SAED images display corresponding positions during first two cycles' discharge/charge process. On the sodiation process, the main peaks of (102), (103), (006), (110), (108), and (116) located at 4°, 4.35°, 4.45°, 6.45°, 7.05°, and 7.85° are not shifted toward the low angles but gradually weakened in intensity until they completely disappear during discharge process in the first cycle, indicating that the crystal lattice is not expanded but amorphizated after Na^+^ insertion. Figure 4c shows the HRTEM and SAED images of the discharge at 1.6 V. The dominant (108), (008), and (100) original planes of CuS were observed in an overall area, and the lattice spacing of 3.05 Å is assigned to the (102) plane of CuS, and a completely amorphous structure appears, with all of the peaks almost disappearing at around 1.0 V after the transition of crystalline state to amorphous state. When discharging to 1.0 V, there are no clear lattice fringes observed in HRTEM and the diffraction patterns of CuS completely disappear in Figure 4d. Then, the appearance of new peaks located at 6.2° could be indexed to the (382) plane of Cu_2_S (PDF#02‐1284) and new peak did not shift until the end of first discharge process. When the electrode fully discharged 0.4 V, the corresponding HRTEM and SAED pictures are presented in Figure 4e. The new diffraction patterns assigned to (220) crystal planes of Cu_2_S with the interplanar spacings of 1.96 Å is clearly seen. This process is irreversible in the charging process that followed. When it is charged to 2.6 V, the peaks of CuS did not reappear demonstrating the proceeding of irreversible conversion reaction in Figure 4b, and the new peak of Cu_2_S shifted to a high angle, indicating that the crystal lattice is shrinked as a consequence of Na^+^ ion deintercalation. In Figure 4f, the lattice space of 3.19 Å is accredited to the (111) plane of Cu_2_S with diffraction of (111) plane when fully charged to 2.6 V. Moreover, amorphous structure could be observed by HRTEM demonstrating the proceeding of amorphization. In the second cycle, the peak of Cu_2_S have shifted toward the low angle in sodiation process, and it have shifted toward the high angle in desodiation process. Importantly, it was found that the crystalline structure of CuS could not be recovered during the second cycle, owing to the formation of irreversible amorphous state. The corresponding HRTEM and SAED images of full sodiation and desodiation are shown in Figure 4g,h. The HRTEM of CuS electrode after 50 and 100 cycles have been supplied in Figure S6, Supporting Information. It could be observed that the amorphous areas also exist in the CuS electrode. The amorphous structure is still well‐maintained during the full charge/discharge process, which is consistent with the in situ SRD results, implying that the irreversible amorphization process is further revealed.

**Figure 4:**
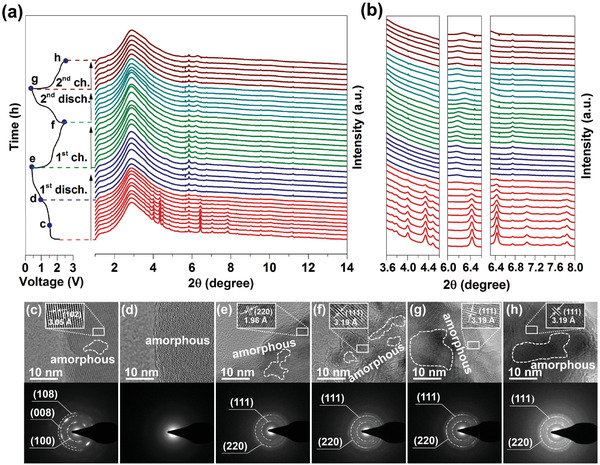
a) In situ SRD patterns of CuS electrode collected at first and second cycle charge–discharge processes between 0.4 and 2.6 V. b) Diffraction peak changes at various 2 theta degrees at first and second cycle charge–discharge states. High‐magnification TEM images and corresponding SAED patterns observed from different charge–discharge states of c) discharge 1.6 V, d) discharge 1.0 V, e) discharge 0.4 V, f) charge 2.6 V in first cycle, and g) discharge 0.4 V in second cycle, and h) charge 2.6 V in second cycle.

From the growth process of hydrangea‐like CuS in Figures 1 and 2, it can be determined that the crystal face preferential orientation growth have occurred along with the (006) crystal face. In the crystal structure of hexagonal CuS, S^2−^ ions have larger ionic radius with stronger electron delocalization compared with O^2−^ ions and the stability of the Cu‐S bond is inferior to that of the Cu—O bond, so that the ordered structure is more easily destroyed. Importantly, the growth characteristics of grain structure along the (006) crystal plane increases the number of Na‐ion intercalation channel, which have promoted to the low polarization of electrodes during charge and discharge process, and the sodium ion is easier to de‐intercalate from Cu‐S clusters after discharge. Meanwhile, the Cu‐S clusters have a low overpotential, illustrating that it is difficult for Cu‐S to form an ordered structure, and finally amorphous structure exists in sodium‐ion storage process.

To get insight into the formed amorphous structure in Na‐CuS system, the DFT calculations, by operating with Vienna Ab‐initio Simulation Package (VASP), were performed to investigate the structural steady state form and the formation energy after Na^+^ insertion.^[^ ^53^ ^]^ The calculations were based on generalized GGA‐PBE (gradient approximation‐Perdew, Burke, and Enzerhof) function for the exchange–correlation potentials and all atoms were relaxed fully until the force acting on each atom was less than 0.02 eV Å^−1^ during the calculation.^[^ ^54^ ^]^ Then, the Na atoms were added into CuS slab model to optimize and the formation energy (*E*_form_) was calculated via the following equation: *E*_form_ = *E*_nNa+CuS_ − *n* × *E*_Na_ − *E*_CuS_, in which *E*_nNa+CuS_, *E*_slab_, and *E*_Na_ denote the total energy of CuS slab with *n* of Na, CuS slab, and free Na atom, respectively. For relaxation of the doped structures, the calculations begin from a symmetry broken structure, with Na moved off its symmetry site. The ions are then allowed to fully relax without symmetry constraints and with symmetry constraints applied. Next, the formation energies of different sodium ions number (*n* = 1, 2, 3, 4, 5, 6, 18, 24, 72, 96) entering the crystal structure of copper sulfide are calculated and their existing forms of phase structure are determined (Figures S7 and S8, Supporting Information). Relaxed GGA geometry of CuS system, 6Na‐CuS system without symmetry constraints, and 6Na‐CuS system with symmetry constraints in top view and side view are presented in **Figure** **5**a–c. The formation energy of *x*Na‐CuS system (*x* = 1–6, 18, 24, 72, 96) is shown in Figure 5d and Table S2, Supporting Information. The crystal structure exhibits atomic occupancy disorder, which means the formation of amorphous structure after the sodium ion embedding in copper sulfide, and the formation energy of 6Na‐CuS system without symmetry constraints is calculated to be −12.4 eV smaller than other *x*Na‐CuS system, indicating that amorphous Na_6_Cu_24_S_24_ is easier to form. Based on the aforementioned results, it could be determined that the form of amorphous structure exists in Na‐CuS system. It is further recognized in theory that sodium ions exist in an amorphous structure, not all of which are in the form of stationary crystals after Na^+^ embedding in the CuS. This result is in good agreement with the previous conclusion of in situ SRD and TEM.

**Figure 5:**
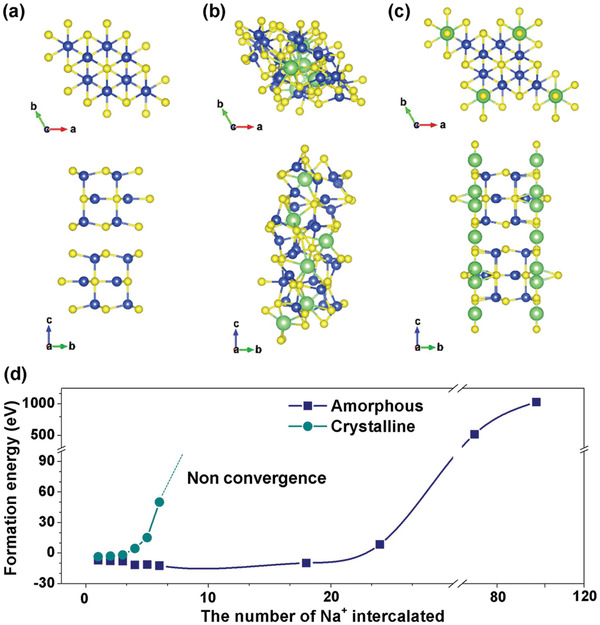
Relaxed GGA geometry of a) CuS system, b) 6Na‐CuS system (without symmetry constraints), and c) 6Na‐CuS system (symmetry constraints) in top view and side view. d) Formation energy of *x*Na‐CuS system (*x* = 1–6, 18, 24, 72, 96).

In summary, we have adopted a simple synthetic method to fabricate hydrangea‐like CuS microsphere with high geometrical symmetry and good sodium storage properties. A possible Ostwald ripening mechanism is proposed to explain the structural evolution from irregular nanosphere to hydrangea‐like CuS microsphere. It delivers a remarkable cycling stability after more than 400 cycles with ≈100% capacity retention and superior rate performance with a discharge capacity of 350.0 mAh g^−1^ at 0.1 A g^−1^. The pseudocapacitive contribution is an important part of excellent rate performance during the charge and discharge processes. More importantly, in situ SRD, TEM, and DFT calculation investigations indicate that the irreversible amorphization process derives from the transition of crystalline state to amorphous state in the sodium‐ion storage process. The amorphous structure could be beneficial to enhance the cycling stability. This discovery and breakthrough will exploit a new way for understanding the phase stability and interphase formation of sulfides during sodium‐ion storage.

## Conflict of Interest

The authors declare no conflict of interest.

## Supporting information

Supporting Information
