# Supplementary material for: Hydrangea‐Like CuS with Irreversible Amorphization Transition for High‐Performance Sodium‐Ion Storage
Source: Adv Sci (Weinh). 2020 Apr 8;7(11):1903279. doi: 10.1002/advs.201903279 (PMC7284207; doi:10.1002/advs.201903279)
Supplement: Supplementary file 1 — Supporting Information [file ADVS-7-1903279-s001.pdf]

Copyright WILEY-VCH Verlag GmbH & Co. KGaA, 69469 Weinheim, Germany, 2020.

ADVANCED SCIENCE
Open Access

Supporting Information

for *Adv. Sci.,* DOI: 10.1002/advs.201903279

Hydrangea-Like CuS with Irreversible Amorphization Transition for High-Performance Sodium-Ion Storage

*Zu-Guang Yang, Zhen-Guo Wu, Wei-Bo Hua, Yao Xiao, Gong-Ke Wang, Yu-Xia Liu, Chun-Jin Wu, Yong-Chun Li, Ben-He Zhong, Wei Xiang, Yan-Jun Zhong, and Xiao-Dong Guo**

Supporting information for

Hydrangea-Like CuS with Irreversible Amorphization Transition for Sodium-Ion Storage

*Zu-Guang Yang, Zhen-Guo Wu, Wei-Bo Hua, Yao Xiao, Gong-Ke Wang, Yu-Xia Liu, Chun-Jin Wu, Yong-Chun Li, Ben-He Zhong, Wei Xiang, Yan-Jun Zhong, Xiao-Dong Guo**

Z.-G. Yang, Z.-G. Wu, Y. Xiao, C.-J. Wu, Y.-C. Li, B.-H. Zhong, Y.-J. Zhong, X.-D. Guo*
School of Chemical Engineering, Sichuan University, Chengdu, 610065, P.R. China

W.-B. Hua
Institute for Applied Materials (IAM), Karlsruhe Institute of Technology (KIT), Hermann-von-Helmholtz-Platz 1, Eggenstein-Leopoldshafen 76344, Germany

G.-K. Wang
School of Materials Science and Engineering, Henan Normal University, Xinxiang 453007, PR China

Y.-X. Liu
The Key Laboratory of Life-Organic Analysis, Key Laboratory of Pharmaceutical Intermediates and Analysis of Natural Medicine, School of Chemistry and Chemical Engineering, Qufu Normal University, Qufu 273165, PR China

W. Xiang
College of Materials and Chemistry &Chemical Engineering, Chengdu University of Technology, Chengdu, 610059, PR China

Experimental Section

Materials syntheses. Synthesis of hydrangea-like CuS microsphere: Copper(II) nitrate hydrate ($Cu(NO_3)_2 \cdot 3H_2O$, 2.0 mmol, 0.4832 g) and Polyvinylpyrrolidone (PVP, 5.4545×10^{-3} mmol, 0.2181g) were dissolved in 70 ml anhydrous ethanol. Then, sulfur powder (S, 4 mmol, 0.1282g) were dispersed in the above solution under ultrasonic dispersion for 30 min. Subsequently, the mixed solution was transferred into a 100 ml Teflon-lined stainless steel autoclave and heated at different temperatures for various times with naturally cooled. The black products were collected by washed with distilled water and absolute ethanol for several times, and then dried at 80 ºC for 12 h in vacuum.

Materials characterizations. Scanning electron microscope (SEM, HITACHI S-4800) were applied to observe the morphology of as-prepared samples. Transmission electron microscopy, selected area electron diffraction (SAED), high-resolution TEM (HR-TEM), high-angle annular dark-field scanning TEM (HAADF-TEM) and elemental mappings were conducted by spherical aberration corrected Transmission Electron Microscope (AC-TEM: FEI Titan G2 60-300) operated at an accelerating voltage of 300 kV to obtain the microstructure of samples. Powder X-ray diffraction with Cu Kα radiation in the 2θ range of 20-70º was used to comfirm crystalline phase of as-prepared material. PDXL software with Rietveld method was used to refine XRD data. X-ray photoelectron spectra (XPS, Thermo Scientific ESCALAB 250Xi) were performed to collect the chemical states of elements. *In situ* high-resolution synchrotron radiation diffraction (SRD) measurements were performed at the P02.1 beamline at DESY, using synchrotron radiation with an energy of 60 keV (λ = 0.20729 Å). The diffraction patterns were collected using a two dimensional detector with sample-to-detector distance of 1810 mm with an exposure time of one minute per pattern.

Electrochemical measurements. CR2032-type coin cells was conducted to evaluate material's electrochemical performance. To fabricate electrodes, a slurry consisting of 75 wt%

active materials, 15 wt.% conductive materials (acetylene black) and 10 wt.% binder (CMC and SBR in ratio of 1:1) with deionized water as solvent was spread on copper foil current collector by a coating machine. Then, the as-prepared electrodes were dried at 120 ºC for 12 h in a vacuum and loading mass of active material was about 2 mg after cutting into disk with a diameter of 1.5 cm. Next, the electrodes were assembled in an argon-filled glove box (Dellis company) using glass fiber (Whatman) as the separator and metallic sodium as reference electrode. The electrolyte was 1.0 M $NaCF_3SO_3$ in diethylene glycol dimethyl ether (DIGLYME). The cells were charged/discharged on a Neware BTS-610 battery test system in the voltage range of 0.4-2.6 V (*vs*. Na/Na^+). And the cells were disassembled at different charged/discharged state to conduct ex-situ TEM measurement. In the transfer process to ex situ test, minimize time exposed to the air of electrodes were required. Cyclic voltammetry (CV) was tested on an electrochemical workstation (LK 9805) from 0.4 to 2.6 V at different scan rate.

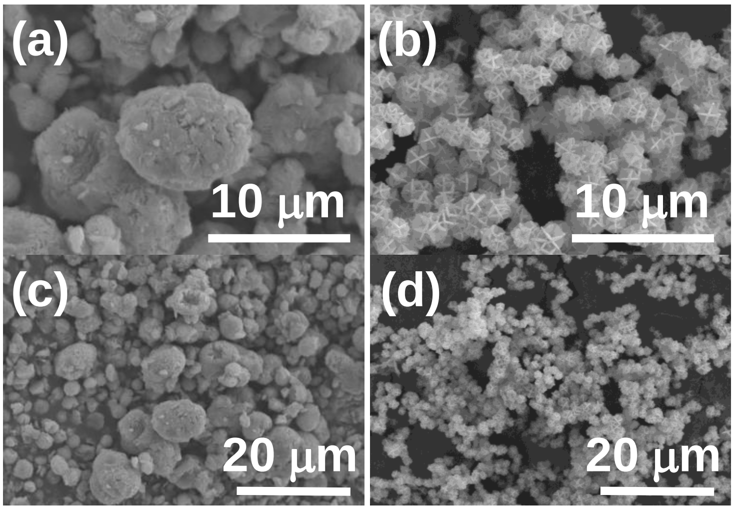

Figure S1. SEM images of CuS prepared: (a, c) without PVP additive, (b, d) added PVP additive.

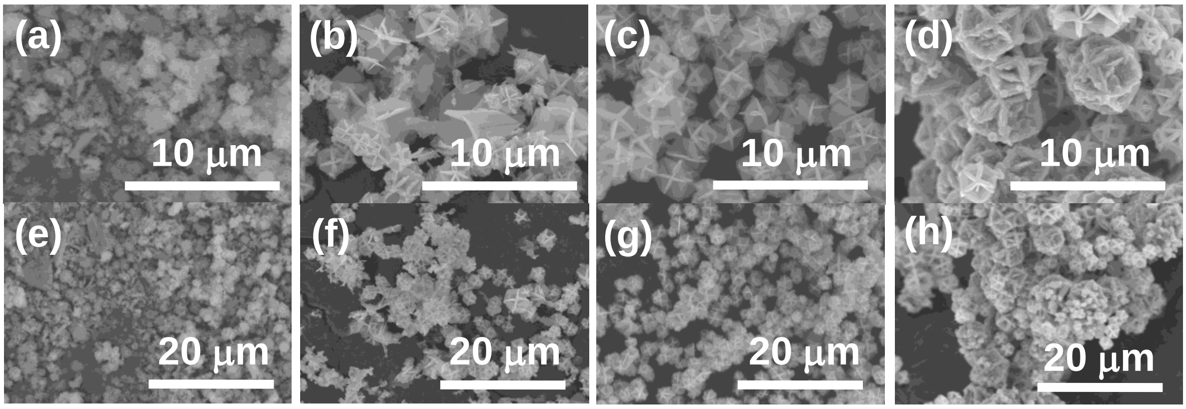

Figure S2. SEM images of CuS prepared at different reaction temperature: (a, e) 100 °C, (b, f) 140 °C, (c, g) 180 °C, and (d, h) 220 °C.

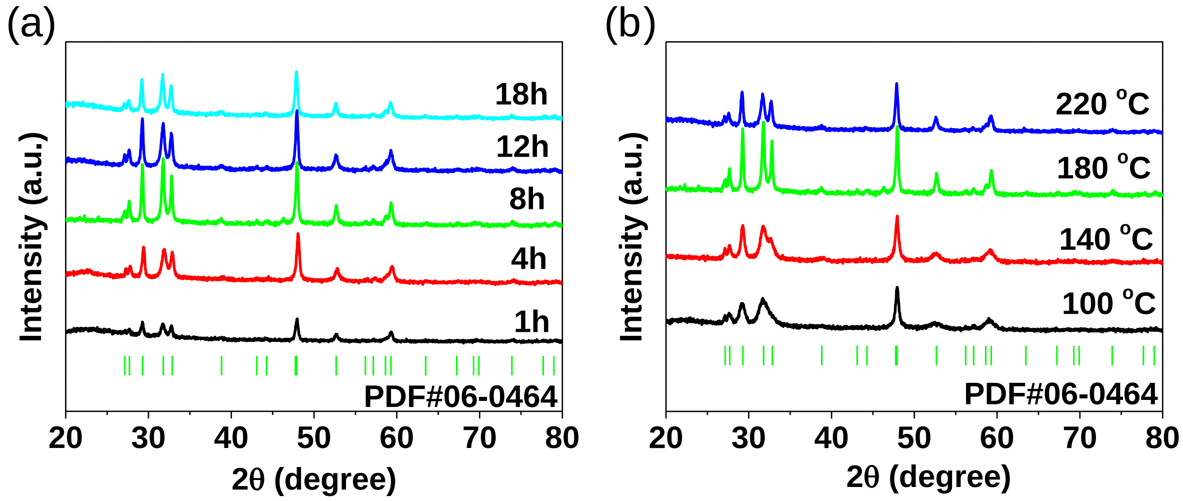

Figure S3. The XRD data of the samples prepared with different conditions: (a) different

times, (b) different temperature.

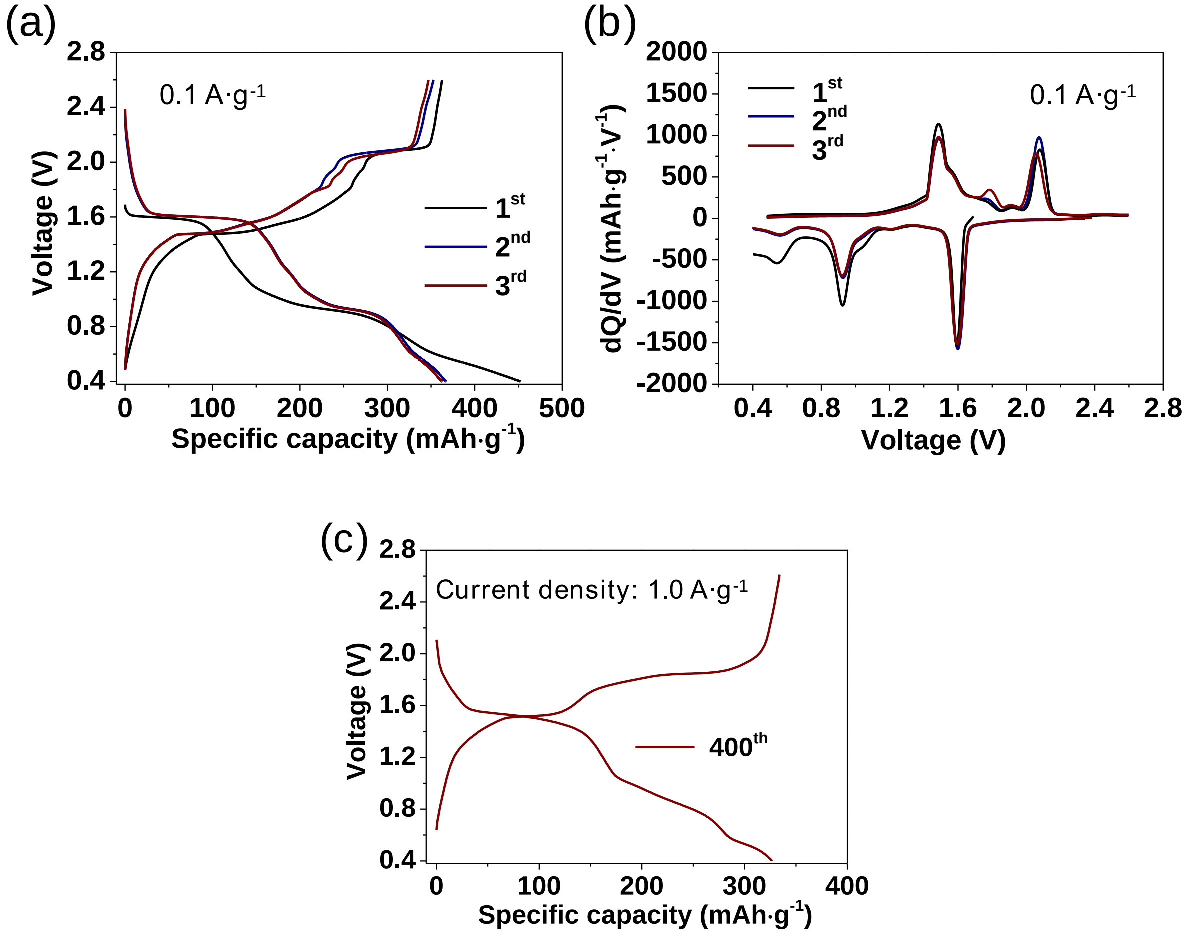

Figure S4. The galvanostatic discharge/charge (GDC) profiles (a) in the first three cycles at 0.1 A·g^{-1} together with the corresponding dQ/dV curves (b) and the GDC curves (c) after 400 cycles at 1.0 A·g^{-1}.

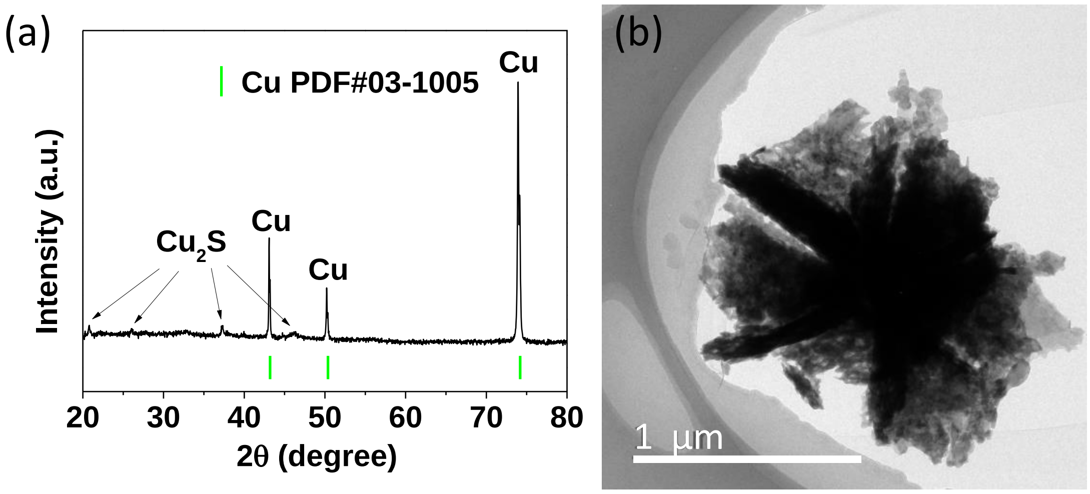

Figure S5. (a) The XRD pattern for the electrodes after 400 cycles and (b) TEM image for the electrodes after 400 cycles.

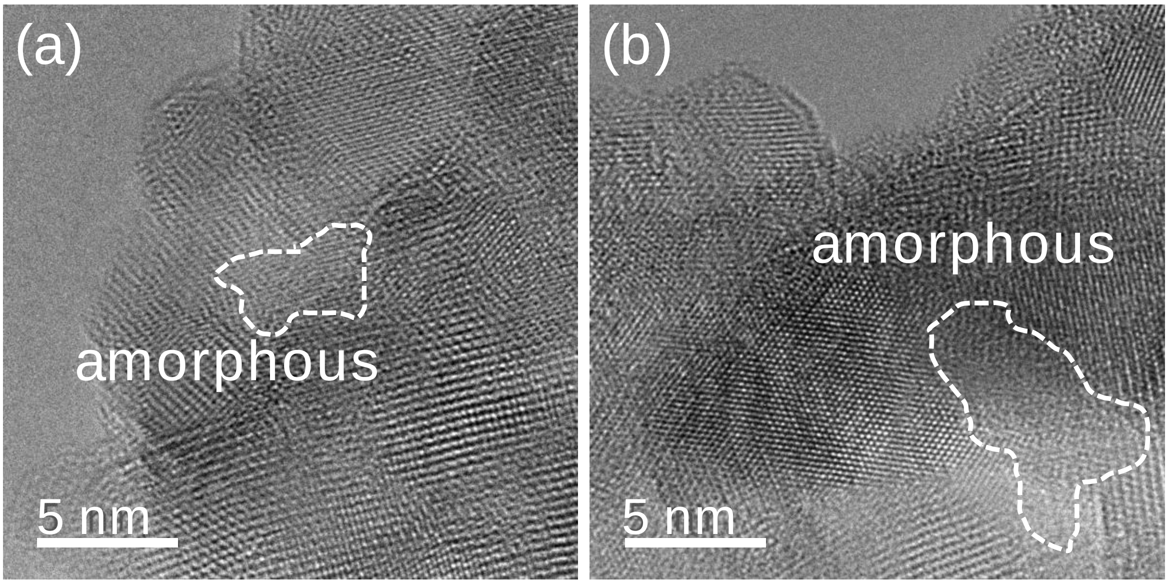

Figure S6. The HRTEM image of CuS electrode after (a) 50 cycles and (b) 100cycles .

DFT calculation

Spin-polarized DFT calculations were performed with Vienna Ab-initio Simulation Package (VASP), using the projector-augmented wave (PAW) method to describe the electron-core interaction.[1] The GGA-PBE was selected for the exchange-correlation potentials.[2] The k point separation in the Brillouin zone of the reciprocal space was chosen as 8 × 8 × 2 for the electronic structures. The atoms were relaxed fully until the force acting on each atom is less than 0.02 eV/Å. All calculations are fully spin polarized.

Covellite (CuS) possesses a hexagonal crystal structure with a space group of P63/mmc (No. 194) at room temperature (Fig. 1). The lattice parameters were calculated and fit to the third-order Birch-Murnaghan equation of states. The calculated results, together with the corresponding experimental data, are summarized in Table I. Our calculated lattice parameters are in agreement with the available experimental and theoretical data.

Then, the Na atoms were added into CuS slab model to optimize and calculate the formation energy (E_{form}), as shown in following Equation.

$$E_{form} = E_{nNa+CuS} - n \times E_{Na} - E_{CuS}$$

where $E_{nNa+CuS}$, E_{slab}, and E_{Na} denote the total energy of CuS slab with n of Na, CuS slab, and free Na atom, respectively.

Table S1. Calculated and experimental lattice parameters of CuS

CuS	*a* = *b* (Å)	*c* (Å)
Calc.	3.79[a]	16.40[a]
Expt.	3.79[b]	16.34[b]

[a] This work.

[b] Reference [3].

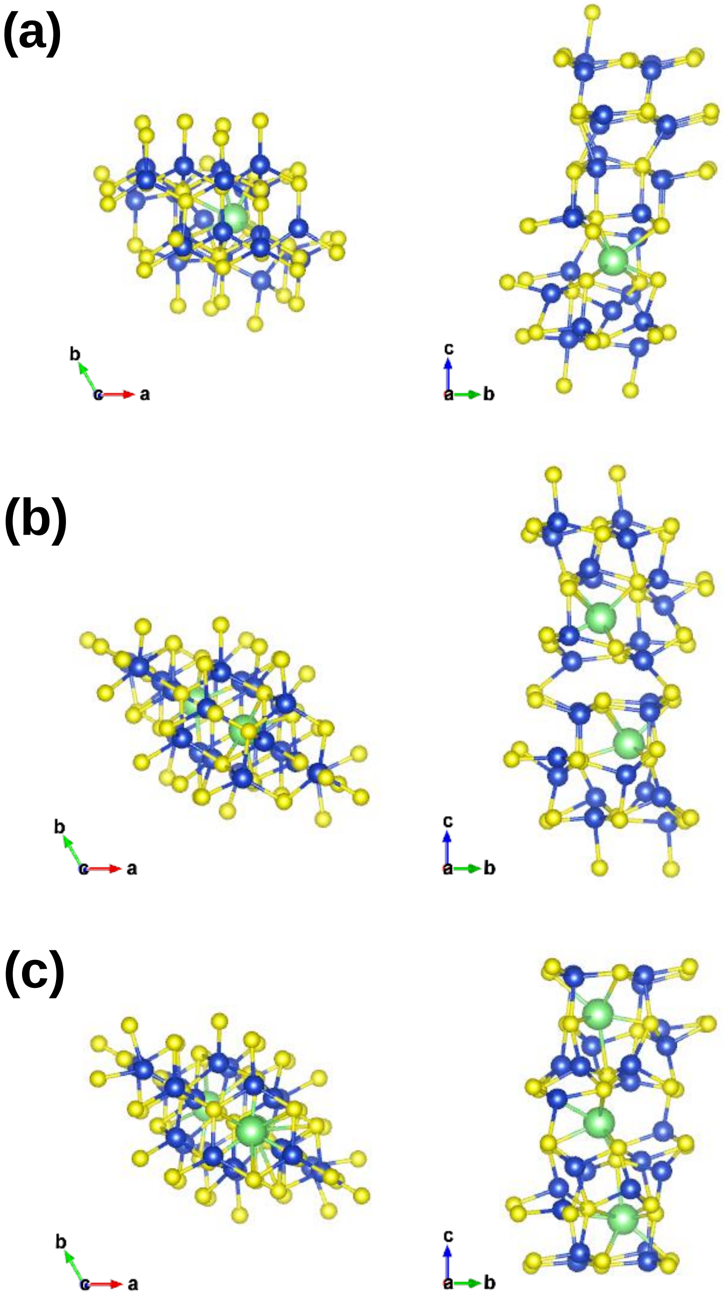

(d)

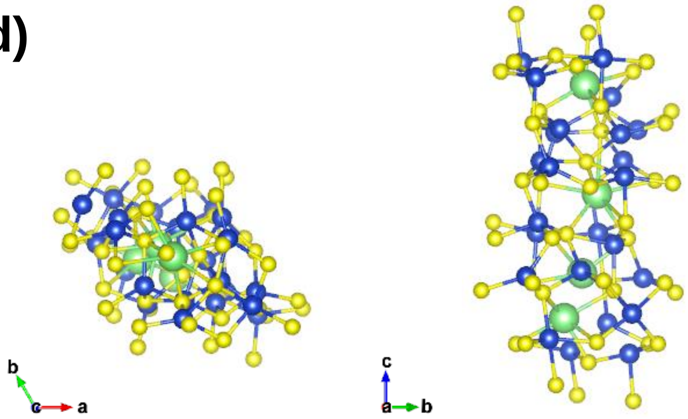

(e)

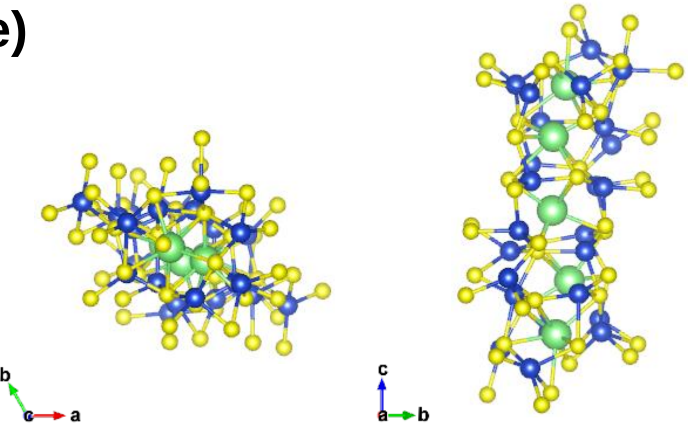

(f)

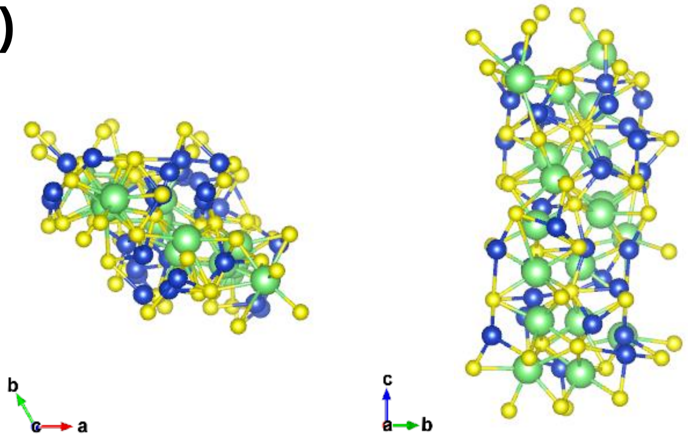

(g)

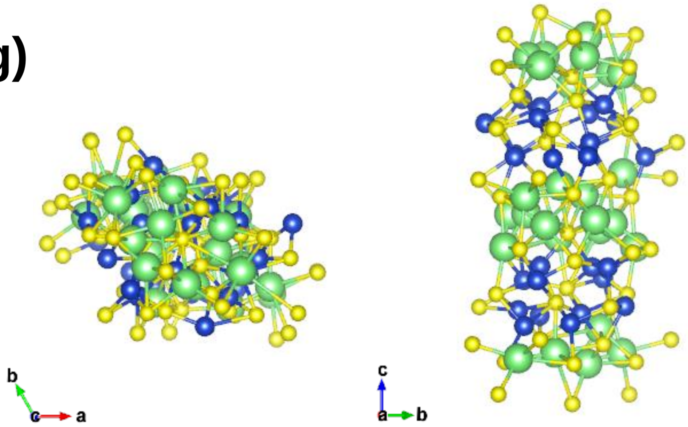

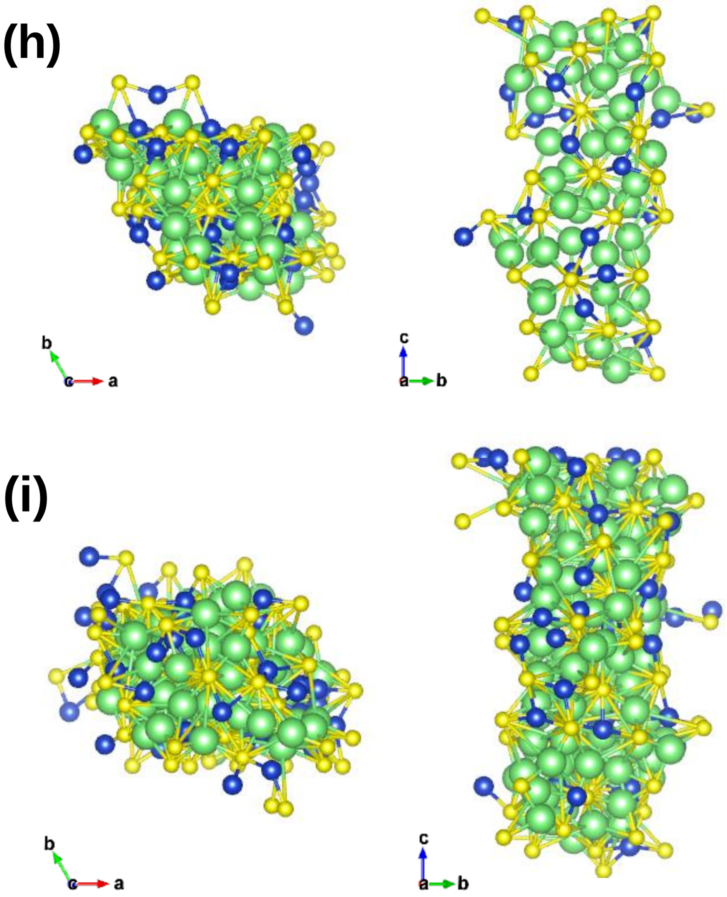

Figure S7. Relaxed GGA geometry of *x*Na-CuS system (*x*=1, 2, 3, 4, 5, 18, 24, 72, 96) without symmetry constraints in top view and side view.

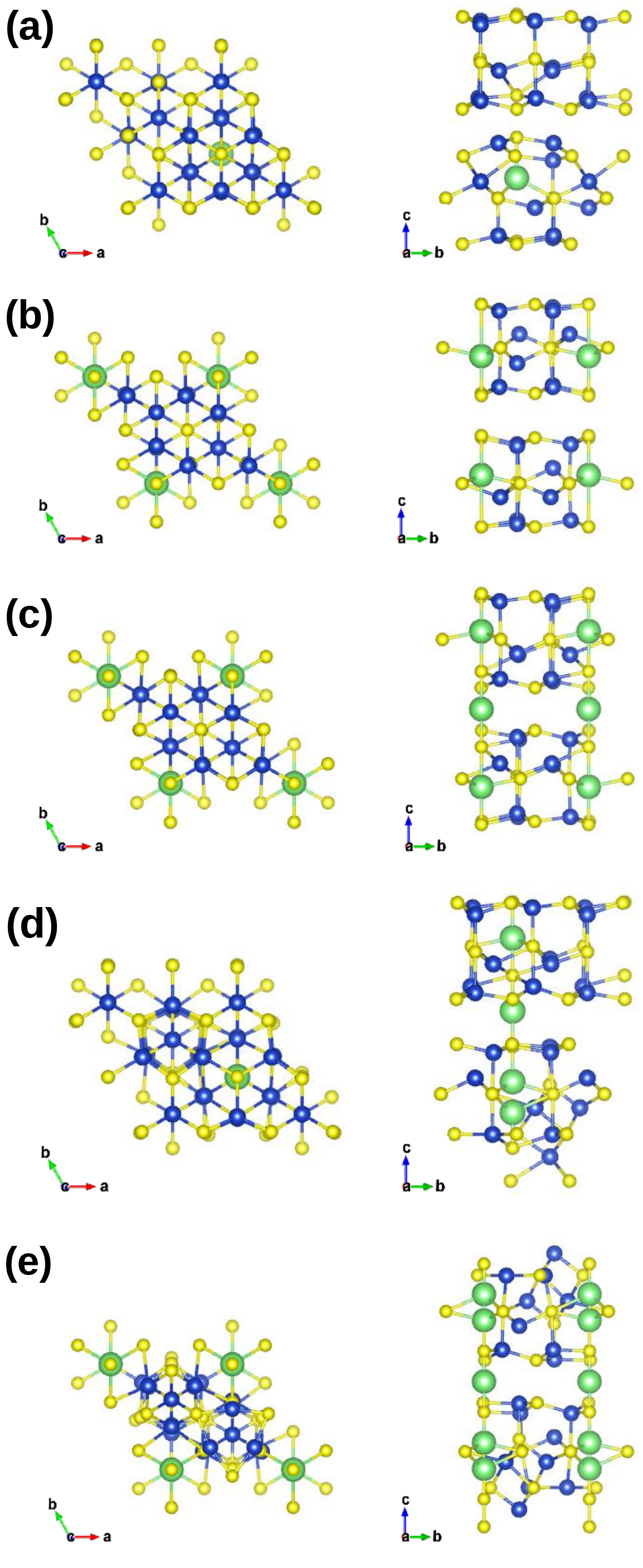
(a)
b
c
a
c
a
b
(b)
b
c
a
c
a
b
(c)
b
c
a
c
a
b
(d)
b
c
a
c
a
b
(e)
b
c
a
c
a
b

(f)

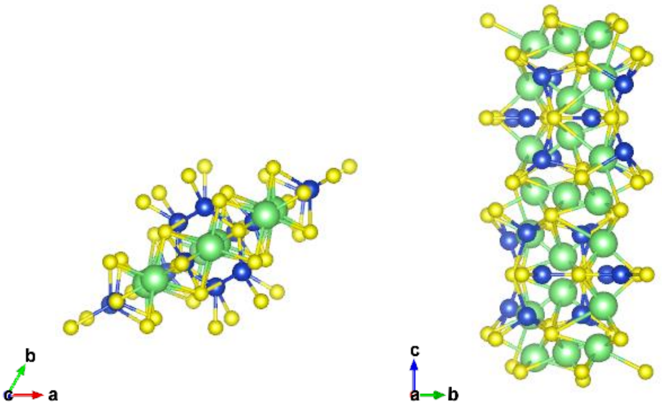

Figure S8. Relaxed GGA geometry of *x*Na-CuS system (*x*=1, 2, 3, 4, 5, 18, 24, 72, 96) with symmetry constraints in top view and side view.

Table S1. Comparison of electrochemical performance for CuS with those of the previous reported works.

Materials	Current density (A g^{-1})	Cycle number	Capacity Retention	Ref
CuS	1.0	400	100 %	This work
CuS-rGO	1.0	450	96.16 %	4
Cu_9S_5	2.0	4000	79%	5
Cu_2S	1.0	400	95.30 %	6
CuS	0.2	200	95.8 %	7
Cu_2S	0.05	20	74.83 %	8
MoS/C	1.0	300	84 %	6
MoS_2	2.0	500	67%	7

Table S2. Formation energy of *x*Na-CuS system (*x*=1-6, 18, 24, 72, 96).

The number of Na^+ intercalated	Formation Energy (eV)	
	with symmetry constraints	without symmetry constraints
1	-7.13	-3.64
2	-7.77	-2.97
3	-7.89	-1.88
4	-11.74	4.50
5	-11.19	15.16
6	-12.40	50.02
18	-9.79	17.02
24	8.47	/
72	512.45	/
96	1036.97	/

References

[1] J. Hafner, J. Comput. Chem. **2010**, *29*, 2044-2078.
[2] J.-P. Perdew, J.-A. Chevary, S.-H. Vosko, K.-A. Jackson, M.-R. Pederson, D.-J. Singh, C. Fiolhais, Phys. Rev. B: Condens Matter. **1993**, *46*, 6671-6687.
[3] Q. Tian, M. Tang, Y. Sun, R. Zou, Z. Chen, M. Zhu, S. Yang, J. Wang, J. Wang, J. Hu, Adv. Mater. **2011**, *3*, 3542-3547.
[4] J. Li, D. Yan, T. Lu. W. Qin, Y. Yao, L. Pan, *ACS Appl. Mater. Interfaces* **2017**, 9, 2309-2316.
[5] Y.-J. Fang, X.-Y. Yu, X.-W. Lou, Angew. Chem. Int. Ed. **2019**, 131, 7826-7830.
[6] M. Boebinger, M. Xu, X. Ma, H. Chen, R. Unocicc, M. McDowell, *J. Mater. Chem. A* **2017**, 5, 11701-11709.
[7] H. Li, Y. H. Wang, J. L. Jiang, Y. Y. Zhang, Y. Y. Peng, J. B. Zhao, *Eletrochimica Acta* **2017**, 247, 851-859.
[8] J. Kima, D. Kima, G. Choa, T. Nama, K. Kima, H. Ryua, J. Ahnb, H. Ahn, *J. Power Sources* **2009**, 189, 864-868.
[9] B. Wang, Y. Xia, G. Wang, Y. Zhou, H. Wang, *Chem. Eng. J.* **2017**, 309 417-425.
[10] T. Sahu, Q. Li, J. Wu, V. P. Dravid, S. Mitra, *J. Mater. Chem. A* **2017**, 5, 355-363.
